# Supplementary material for: Review of Canine Lymphoma Treated with Chemotherapy—Outcomes and Prognostic Factors
Source: Vet Sci. 2023 May 11;10(5):342. doi: 10.3390/vetsci10050342 (PMC10224423; doi:10.3390/vetsci10050342)
Supplement: Supplementary file 1 [file vetsci-10-00342-s001.zip › vetsci-2357317-supplementary.pdf]

Supplemental Materials:

Table S1: Prognostic variables that were reported in less than 10 papers.

NA – not applicable as not included in relevant paper.

		**Papers included**			**% Of papers where prognostic for:**	
**Variable included in analysis**	**Number of papers**	**Both remission and survival**	**Remission only**	**Survival only**	**Remission**	**Survival**
Patient Characteristics:						
Anorexia	2	2	0	0	0.0	50.0
Fever	1	1	0	0	0.0	0.0
Cachexia	1	1	0	0	0.0	0.0
Glasgow performance score	1	1	0	0	0.0	100.0
Geographic locality	1	0	1	0	100.0	NA
Lymphoma Characteristics:						
Cell size	3	2	0	1	50.0	100.0
Grade	6	4	1	1	0.0	20.0
Histology	8	4	1	3	20.0	42.9
Mitotic count	1	0	0	1	NA	100.0
Laboratory findings:						
Albumin	3	3	0	0	66.7	66.7
LDH	4	3	0	1	33.3	0.0

Neutrophils (Number or ratio)	7	7	0	0	28.6	28.6
Monocytes (Number of ratio)	6	6	0	0	33.3	16.7
ALP	3	0	3	0	0.0	NA
T-reg	1	1	0	0	100.0	100.0
Bilirubin	1	0	1	0	0.0	NA
Urea	1	1	0	0	0.0	0.0
Coagulation	1	1	0	0	100.0	100.0
Ferritin	1	0	0	1	NA	100.0
Treatment parameters:						
Toxicity	5	4	1	0	20.0	25.0
Dose Modification	6	5	1	0	16.7	40.0
Weight change during treatment	2	2	0	0	50.0	50.0

Table S2: Papers that included a molecular, genetic, immunohistochemical or other biomarker as a prognostic factor analysed by multivariate analysis.

**Paper**	**Factors included**	**Prognostic for remission**	**Prognostic for survival**
Aresu et al 2021 [72]	PD-L1, PD1, CD8A, Ki67	PD-L1, PD-1	PD-L1
Bergman et al 1996 [77]	P-glycoprotein	P-glycoprotein	P-glycoprotein
Deravi et al [91]	T-cell immunophenotypes	CD4, CD8, MHCII	CD4, CD8, MHCII

Fujiwara-Igarashi et al 2014 [97]	p14, p15, p16	NA	p16
Garnica et al 2020 [98]	microRNA from small extravesicular vesicles (SEV)	NA	SEV concentration, microRNA
Hahn et al 1994 [195]	Karyotype	Trisomy chromosome 13	Trisomy chromosome 13
Kiupel et al 1999 [190]	AgNOR	AgNOR	AgNOR
Lee et al 1996 [191]	P-glycoprotein		P-glycoprotein
Pinheiro et al 2014 [192]	T_{reg}, MHCII	T_{reg}, MHCII	T_{reg}
Poggi et al 2017 [124]	K-67		Ki-67
Rao et al 2011 [193]	MHCII, CD34, CD21, CD5	MHCII	MHCII
Vail et al 1996 [194]	Potential doubling time, AgNOR, PCNA, DNA ploidy	Potential doubling time, AgNOR	AgNOR
Von Euler et al 2004 [195]	Serum thymidine kinase		Serum thymidine kinase
Williams et al 2008 [196]	CD3, CD4, CD5, CD8, CD18, CD21, CD34, CD34, MCHII	NA	CD34

NA – remission or survival not analysed.

Table S3: Papers evaluating treatment protocol variations.

**Paper**	**Treatment parameter analysed**	**Prognostic?**
Aresu et al 2021 [72]	Addition of a vaccine to CHOP protocol	Yes
Boye et al 2021 [196]	Experimental drug (F14512) or etoposide	No
Deravi et al 2017 [91]	Combination versus single agent in T-cell	No
Goodman et al 2016 [100]	Substitution of mitoxantrone in VELCAP-TSC	No
Hahn et al 1994 [195]	Doxorubicin versus epirubicin	No
Hosoya et al 2007 [5]	COAP versus UWM-19	Yes
Lautscham et al 2017 [166]	Maintenance versus no maintenance	No
Lee et al 1996 [191]	CHOP-like combination versus doxo or doxo/vinc	No
Miller et al 2009 [197]	CHOP versus doxorubicin	Yes
Mutsaers et al 2002	Doxo versus doxo/piroxicam	No
Pinheiro et al 2014 [192]	CHOP versus other protocols that did not include doxorubicin	Yes
Rao et al 2011 [193]	CHOP versus single agent versus pred	Yes
Rassnick et al 2010 [64]	CHOP versus CHOP/CCNU/MOPP	Yes
Romano et al 2016 [169]	CHOP versus others	No

Siedlecki et al 2006 [128]	Drug substitutions in CHOP-like protocol	No
Simon et al 2008 [35]	CHOP versus doxo	No
Zandvliet et al 2013 [135]	CHOP with versus without prednisolone	No

Table S4: Summary findings from the papers that only included univariate analysis. Px = prognosis.

**Factor**	**Px – Remission**	**Px – Survival**	**Px – Both**	**Not Px – Remission**	**Not Px - survival**	**Not Px - both**	**Not included**
**Studies with Univariate analysis (n=52) – studies (cases) (total cases = 2958)**							
Age	0	1(73)	1 (31)	2 (82)	5(258)	5(442)	38(2072)
Weight	0	0	0	2 (82)	2(78)	7(513)	41(2285)
Breed	0	2(194)	0	0	0	0	50(2764)
Sex	1 (88)	0	2 (245)	2 (82)	5(258)	8(479)	34(1806)
Neuter	0	0	0	0	0	0	52 (2958)
Anatomic location[a]	0	1 (26)	0	1 (46)	0	1 (24)	48(2851)
Stage	3 (123)	3 (246)	1 (43)	3(153)	2 (96)	12 (997)	28(1300)
Substage	4 (141)	4(373)	5 (470)	2(130)	2(78)	5(470)	31(1629)
Immunophenotype	3(145)	1 (112)	2 (201)	0	3(95)	2 (161)	41(2244)
Prior prednisolone	1 (28)	0	0	0	1 (62)	0	50(2868)
Anaemia	0	0	0	0	1(121)	4(130)	47(2707)
Thrombocytopenia[b]	0	1(24)	0	0	1(121)	1(31)	49(2782)
Neutrophils[c]	2(115)	0	0	0	0	1 (24)	49(2819)
Monocytes[c]	0	0	0	0	0	0	52 (2958)
Hypercalcaemia	0	0	1 (97)	2 (105)	0	5(337)	44(2419)
Hypoalbuminaemia	1 (28)	0	0	0	0	1(18)	50(2912)

Response	0	3 (302)	3 (165)	1 (46)	0	1 (37)	44(2408)
Toxicity	0	0	1 (50)	0	0	2 (142)	49(2766)
Dose modification	1 (62)	0	1 (130)	0	0	1 (104)	49(2662)
Anorexia	0	0	0	0	0	0	52(2958)
Histology	0	0	2 (77)	0	0	2(60)	48(2821)
Grade	0	0	0	0	0	2 (153)	50(2805)
Coagulation	0	1 (27)	0	0	0	0	51(2931)

[a] – 1 study (11 cases) only included rectal lymphoma and compared to literature

[b] – 1 study (24 cases) of T-cell lymphoma showed thrombocytopenia to be a positive prognostic factor for survival

[c] - neutrophils and monocytes include both absolute numbers and ratios
